# Surface Electromyography and Gait Features in Patients after Anterior Cruciate Ligament Reconstruction

**DOI:** 10.1111/os.14256

**Published:** 2024-10-05

**Authors:** Xipeng Wu, Hao Zhang, Hongxing Cui, Wenbin Pei, Yixuan Zhao, Shanshan Wang, Zhijie Cao, Wei Li

**Affiliations:** ^1^ School of Rehabilitation Medicine Binzhou Medical University Yantai China; ^2^ Department of Rehabilitation Binzhou Medical University Hospital Binzhou China

**Keywords:** Anterior Cruciate Ligament Reconstruction, Gait, Lower Limbs, Surface Electromyography

## Abstract

**Objective:**

An important reason for the poor recovery of anterior cruciate ligament (ACL) injuries is the poor recovery of muscle function. Therefore, we used surface electromyography (sEMG) and gait analysis to explore the muscle activation patterns and gait characteristics between lower limbs under different exercise states in patients, following anterior cruciate ligament reconstruction (ACLR).

**Methods:**

Forty‐one adults with unilateral ACL injuries in Binzhou Medical University Hospital from October 2022 to June 2023 were allocated to three groups according to the time after ACL reconstruction: group A (≤3 months, 16), group B (3 months–1 year, 13), and group C (>1 year, 12). Patients were tested by sEMG and gait, while straight leg raising (SLR), walking at normal speed, fast walking, and walking up and down the stairs. Two related sample tests were performed for the normalized root mean square (RMS) values and gait parameters.

**Results:**

Muscle function changes varied in different training tasks. The RMS value of the involved side was more than the uninvolved side in biceps femoris and semitendinosus of group A (*p* < 0.010), and for the bilateral rectus femoris (RS), vastus medialis (VM), and vastus lateralis in group B, only the comparison of the RS was significant in group C during fast walking and going up and down the stairs. The ground impact (0.90 [0.63, 1.33] vs. 0.71 [0.43, 1.02], *p* = 0.035) of the uninvolved side was significantly decreased compared to those of the involved side in patients with ACLR when going down the stairs.

**Conclusion:**

Different muscles need to be focused on at different stages of the postoperative period. sEMG and gait analysis can guide the development of a rehabilitation program.

## Graphical abstract

The purpose of this study is to conduct sEMG and gait analysis to explore the muscle and gait characteristics of anterior cruciate ligament reconstruction (ACLR) patients. It provides a basis for the development of individualized rehabilitation programs.

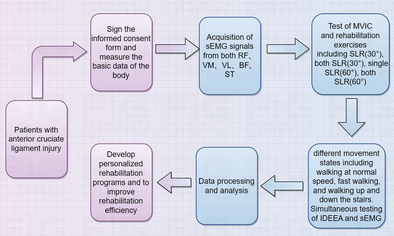

## Introduction

Anterior cruciate ligament (ACL) injuries occur frequently during sports activities and are one of the most common knee injuries in athletes, severely affecting the athletic and work capacity of afflicted young adults.^1^, ^2^, ^3^ More than 250,000 ACL injuries occur annually in the United States, and the preferred treatment for ACL injuries is anterior cruciate ligament reconstruction (ACLR) surgery, which is performed on approximately 65% of patients.^4^, ^5^ However, the current recovery rate of motor ability in patients after ACLR is not satisfactory, which has been shown to be related to the current characterization of muscle activation.^6^

Surface electromyography (sEMG) is a popular research tool in exercise and rehabilitation science that is widely used to understand posttraumatic changes in neuromuscular function.^7^ This technique quantifies muscle contraction and diastolic activity^8^ from recorded bioelectrical signals generated from neuromuscular activity from one or more pairs of electrodes placed on the skin.^9^ Thus, sEMG is a unique tool for monitoring the functional neuromuscular system during movement.^10^ The sEMG signal reflects the characteristics of muscle function and can provide information about muscle activity to clinicians, thereby facilitating the development of a customized rehabilitation program for patients with muscle dysfunction.^11^ Compared with traditional needle electromyography, sEMG has the advantages of being noninvasive, portable, and wearable, which can assess the muscle motor function in real time and dynamically. When combined with gait analysis, it can clarify the relationship between different muscle activities and the temporal phases of the gait cycle, which can provide more bases for the evaluation of diseases and the formulation of treatment plans. When combined with clinical assessment, it provides timely insight into the patient's condition and should enable clinicians to understand which muscles may be weak and not play a significant role in activities^12^, thereby helping clinicians to use this information as a foundation to develop specific postoperative rehabilitation programs.

The lack of biomechanical basis is one of the major reasons for the imprecision of current rehabilitation programs, which affects the effectiveness of rehabilitation, so we intend to explore the sEMG and gait features after ACLR. The rehabilitation protocols currently in use were primarily developed based on injury duration and aimed to improve the motor function of the patient and reduce the risk of reinjury.^5^ However, the process of injury recovery is complex and influenced by several factors. Rehabilitation following ACLR should not be developed within a strict time frame. Instead, it should be decided on objective bases, such as sEMG data, to develop progressive criteria with graded increases in activity difficulty.^13^ Currently, there is a lack of consensus on the standard of rehabilitation that should be achieved before returning to sports^14^. Professional physicians, feedback techniques, and qualitative motor analyses are essential for completing different tasks^15^. Therefore, the aims of this study are as follows: (i) to discover the sEMG and gait features of ACLR patients at different stages and exercise states and to reveal the sEMG and gait patterns of lower limb muscle after ACLR; (ii) to reveal the effects of different rehabilitation training tasks on muscle activation and gait; and (iii) to develop objective and quantitative personalized rehabilitation programs based on sEMG and gait and to help reduce the incidence of new injuries and lower the risk of reinjury.

## Methods

### Participants

Forty‐one eligible patients from October 2022 to June 2023 who underwent primary ACLR were enrolled in the present study. The patients were divided into three groups according to the time since surgery: group A (≤3 months), group B (3 months–1 year), and group C (>1 year). The basic information of all participants is shown in Table 1. This study was approved by the local ethics committee (Protocol 2022‐G29‐01).

**TABLE 1:** The general characteristics of different groups.

	Group A	Group B	Group C
*N*	16	13	12
Age (years)	30.56 ± 10.61	33.54 ± 10.05	30.58 ± 10.46
Height (cm)	175.75 ± 8.91	172.62 ± 8.88	176.33 ± 8.98
Weight (kg)	81.81 ± 16.09	76.77 ± 14.25	76.67 ± 18.93
Body mass index (kg/m^2^)	26.35 ± 4.33	25.55 ± 2.56	24.40 ± 4.18

The inclusion criteria were as follows: (i) no significant osteoporosis or joint degeneration, as confirmed by preoperative examination; (ii) complete rupture of the ACL as diagnosed using magnetic resonance imaging (MRI); and (iii) simple ACL injury with or without one degree of cartilage injury and without meniscal injury.

The exclusion criteria were as follows: (i) patients with only partial ACL injury diagnosed by MRI; (ii) patients with significant osteoporosis or previous knee injury, history of deep vein thrombosis or disorders of the coagulation system, systemic diseases affecting physical function, and other diseases; (iii) patients with a combination of diseases that may affect postoperative exercise; and (iv) patients with a second degree or higher cartilage or meniscal injury.

### Test Procedure

**FIGURE 1:**
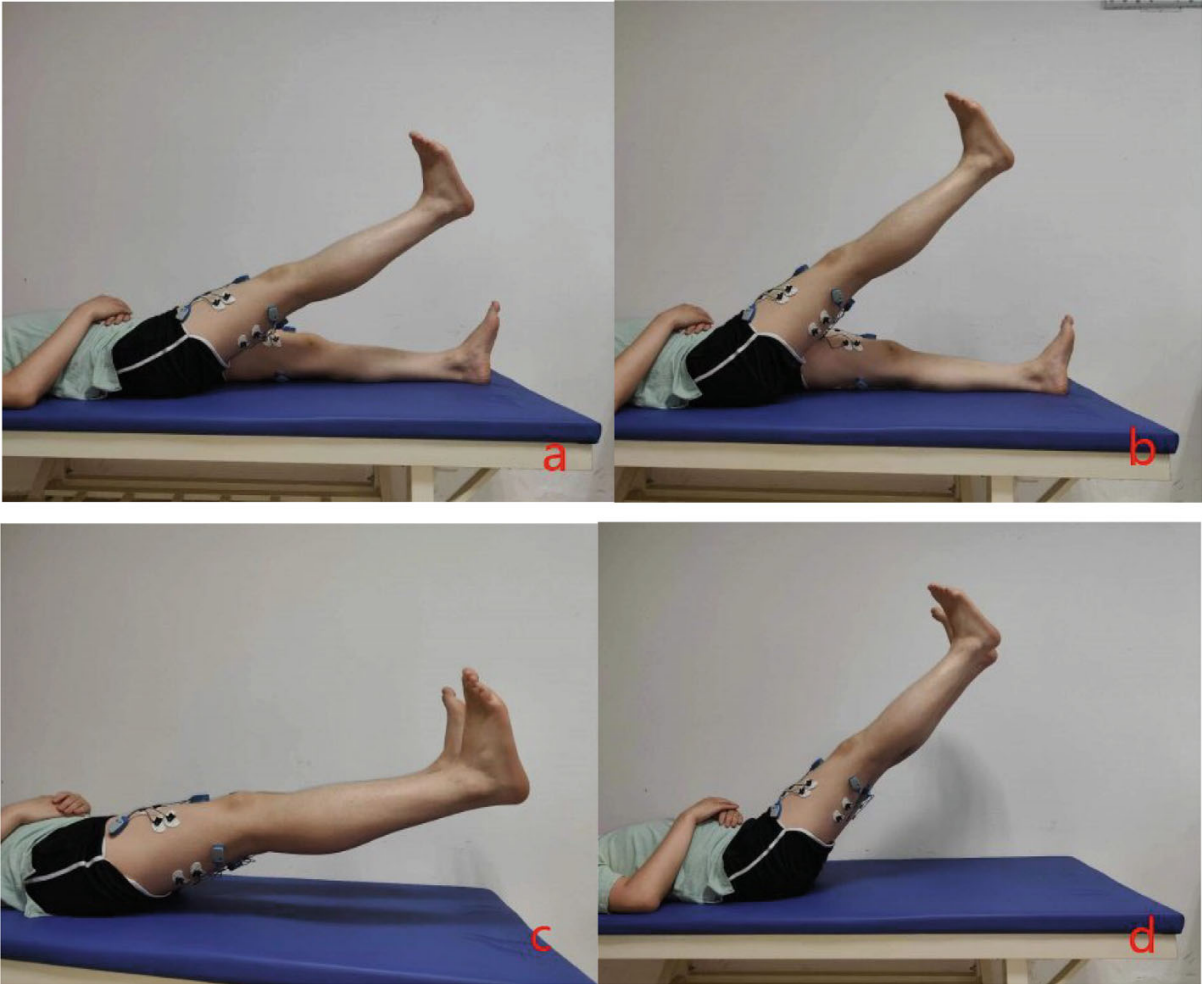
The tasks performed during the experiment include single SLR (30°) (A), single SLR (60°) (B), both SLR (30°) (C), both SLR (60°) (D).

The participants were introduced to the procedure and test protocol on the test day. The test protocol comprised five procedures as follows (in chronological order): (a) measurement of anthropometric data (age, height, weight, and determining dominant leg), (b) positioning of bipolar sEMG electrodes, (c) standardized warm‐up procedure, (d) test of maximum voluntary isometric contraction (MVIC), (e) rehabilitation exercises including single straight leg raising (SLR) (30°), both SLR (30°), single SLR (60°), both SLR (60°) (Figure 1A–D) tests, and (f) different movement states including walking at normal speed, fast walking, and walking up and down the stairs. Simultaneous testing of IDEEA (IDEEA, MiniSun, LLC, Fresno, CA, USA) and sEMG (Noraxon Ultium EMGSystem, Scottsdale, USA) was performed during different movements.

### Data Acquisition

#### Maximum Voluntary Isometric Contraction (MVIC)

All participants were evaluated at the same location by the same examiner and wore athletic shorts and standard shoes during the test. All subjects performed a 7‐min warm‐up session, which involved running, side running, deep squats, and double‐leg jumps.

The patient was seated on the edge of the bed with the knee at 90°, and resistance was applied to the ankle for knee extension to obtain the MVIC of the knee extensors. The MVIC of the knee flexor was obtained with the patient lying prone on the examination bed with the knee flexed at 10° and applying resistance to the ankle to perform maximum isometric knee flexor contraction.^16^

All MVIC tests lasted for 4 s to allow maximal muscle activity, and strong verbal encouragement was provided to the subjects. Three MVIC trials were performed for each muscle with a 30‐s rest between trials to avoid fatigue accumulation.

#### sEMG Measurement

Bipolar sEMG electrodes were placed over the bilateral rectus femoris (RF), vastus medialis (VM), vastus lateralis (VL), biceps femoris (BF), and semitendinosus (ST) muscles. All signals were sampled at 1200 Hz with a 20–500 Hz bandpass filter. Before electrode placement, the subject's body surface hair was shaved, skin keratin was polished using fine gauze, and body surface sebum was cleaned with 70% ethanol. The tasks performed during the experiment include MVIC, single SLR (30°), both SLR (30°), single SLR (60°), both SLR (60°), walking at normal speed, fast walking, and walking up and down the stairs.

#### Gait Measurement

The Intelligent Device for Energy Expenditure and Activity (IDEEA, MiniSun, LLC, Fresno, CA, USA) was applied to monitor physical activity and measure gait parameters. The participants were asked to wear this device during different movement states, including walking at normal speed, fast walking, and walking up and down the stairs.

### Data Analysis

#### sEMG Analysis

All raw sEMG signals were processed using custom MATLAB (MathWorks, Inc. Natick, MA, USA) algorithms. First, a fourth‐order high‐pass filter was applied with a cutoff frequency of 10 Hz, after which a moving (1‐ms steps) root‐mean‐square filter with a 30‐ms time constant was applied. After a standardized warm‐up program, three MVIC trials for the hamstring and quadriceps muscles were performed (a detailed description is provided later). The peak sEMG amplitude obtained during MVIC from each muscle (identically filtered) was used to normalize the peak sEMG values obtained during the respective exercises.

Maximum values were extracted for normalization. Furthermore, the root mean square (RMS) was recorded during the sustained motor task using 1‐s windows of the sEMG signals and normalized to the maximum RMS values from the maximum voluntary contraction tests.

#### Gait Analysis

Gait data were transferred to a computer and analyzed by ActView3 (MiniSun, USA). Single leg support time, swing power, ground impact, and pulling acceleration were used to evaluate when patients walking at normal speed, fast walking, and walking up and down the stairs.

### Statistical Analysis

All statistical analyses were performed using Statistical Package for the Social Sciences (SPSS) software (version 26.0, IL, USA). The Shapiro–Wilk test was applied to test the normality of all data. However, all data failed the normality tests. Therefore, two related sample tests were performed for the normalized RMS values and gait parameters. Differences were considered statistically significant at *p* < 0.050.

## Results

### sEMG Measurement Results

**FIGURE 2:**
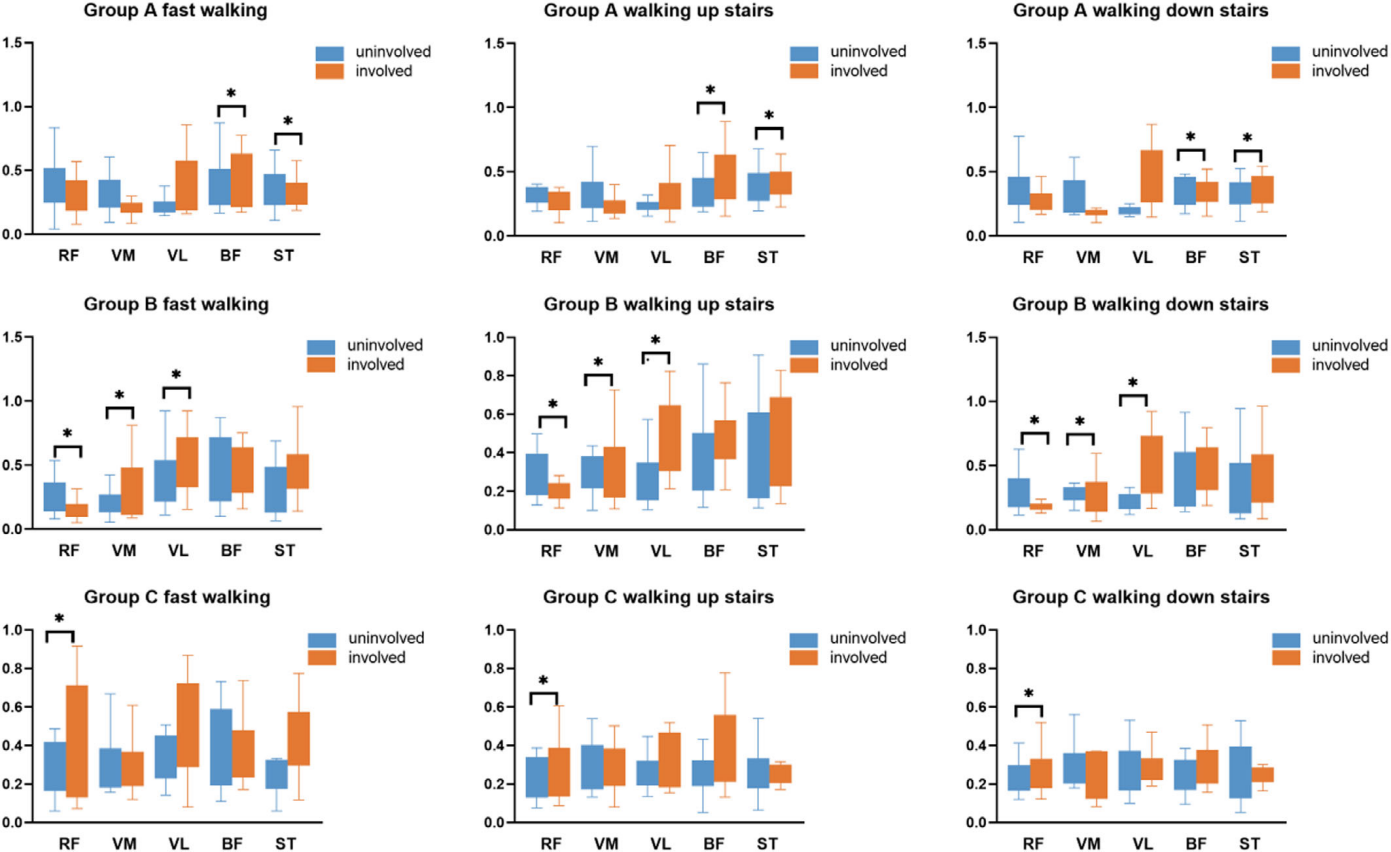
The RMS value of the muscles in bilateral limb of different training methods. In the special exercise state, the activation of the affected side of the BF and ST in group A was greater than that of the unaffected side, the quadriceps in group B was larger than the unaffected side, and the RF in group C was greater than the unaffected side.

This study found that the muscle activation of the involved limbs was greater than that of the uninvolved limbs. During normal‐speed walking, the muscle activation of the RF (*p* = 0.023), VM (*p* = 0.002), VL (*p* = 0.003), BF (*p* = 0.003), and ST (*p* = 0.017) on the involved are greater than the uninvolved limbs in group A, while in group B, the involved side of RF (*p* = 0.002) and VM (*p* = 0.011) has more muscle activation than the uninvolved side, and in group C, RF (*p* = 0.010) and ST (*p* = 0.019) has more muscle activation on the involved side than on the uninvolved side. We further found a commonality in the comparisons between the involved and the uninvolved limbs in the motion states of fast‐speed walking and going up and down the stairs, and the comparison of BF and ST was significant in group A (*p* < 0.010), while in group B, the differences were significant for RF, VM, and VL (*p* < 0.010); and in group C, only the comparison of RF (*p* < 0.010) was significant (Figure 2).

**FIGURE 3:**
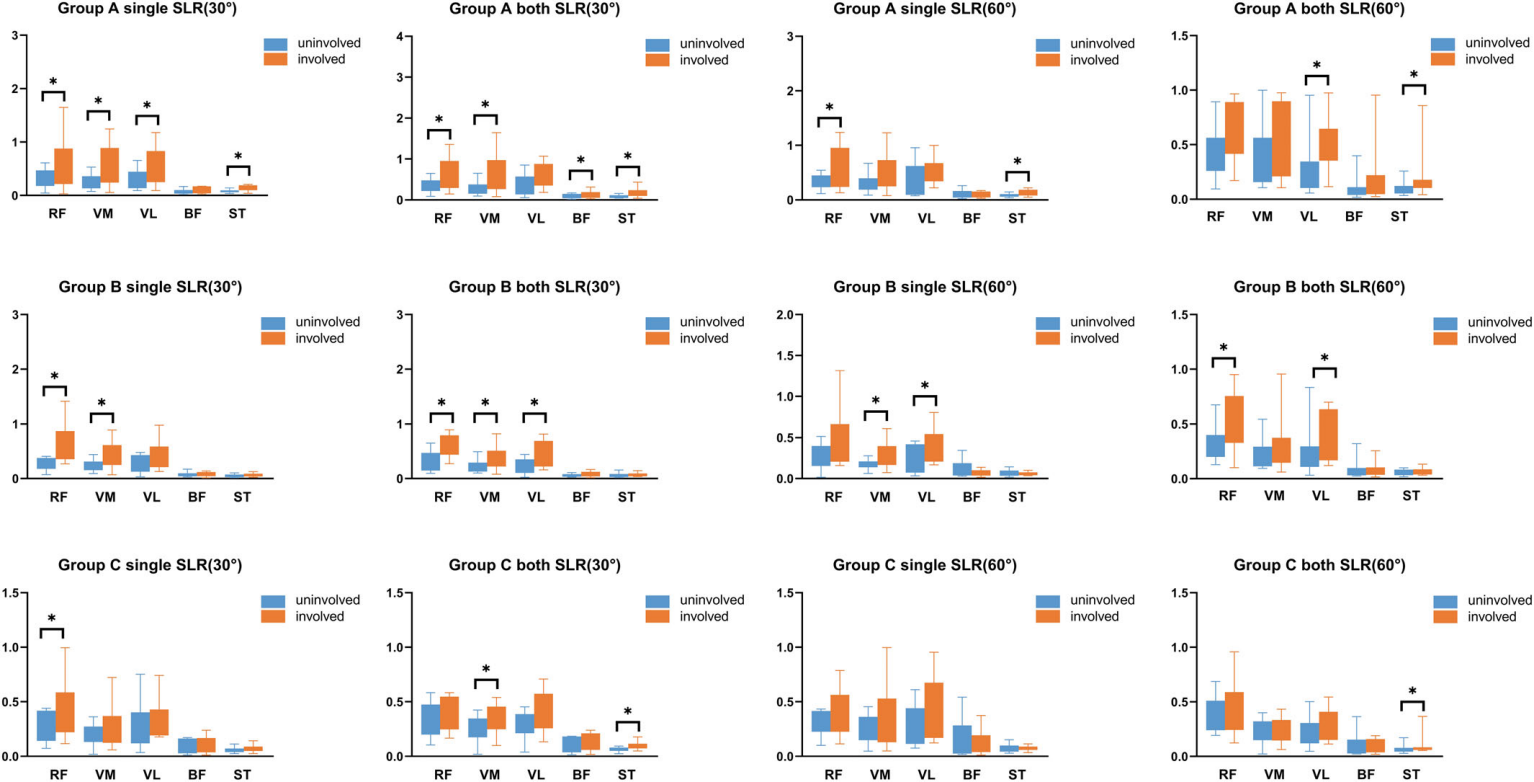
The RMS value of the muscles in the bilateral limb of different training tasks. For people in group A, SLR (30°) is a better way of training than SLR (60°). For those in groups A and B, both SLR (30°) is a better way to train the RF and VM. For group C, both SLR (30°) were only effective for training the VM. The single SLR (60°) is more suitable for those in group B to train the VM as well as the VL. Both SLR (60°) can achieve a better training effect for the VL in group A and group B.

For different training tasks, muscle activation was different in each group. At single SLR (30°), the muscle activation of the RF (*p* = 0.039), VM (*p* = 0.008), VL (*p* = 0.020), and ST (*p* = 0.006) on the involved are greater than the uninvolved limbs in group A, and in group B the involved side of RF (*p* = 0.016) and VM (*p* = 0.046) has more muscle activation than the uninvolved side, only RF (*p* = 0.019) has more muscle activation on the involved side than on the uninvolved side in group C. At both SLR (30°), the muscle activation of the RF (*p* = 0.034), VM (*p* = 0.026), BF (*p* = 0.030), and ST (*p* = 0.004) on the involved are greater than the uninvolved limbs in group A, and in group B the involved side of RF (*p* = 0.004), VM (*p* = 0.028), and VL (*p* = 0.019) have more muscle activation than the uninvolved side, VM (*p* = 0.034), and ST (*p* = 0.005) has more muscle activation on the involved side than on the uninvolved side in group C. At single SLR (60°), the muscle activation of the RF (*p* = 0.039) and ST (*p* = 0.010) on the involved are greater than the uninvolved limbs in group A, and in group B the involved side of VM (*p* = 0.028) and VL (*p* = 0.028) has more muscle activation than the uninvolved side. At both SLR (60°), the muscle activation of the VL (*p* = 0.030) and ST (*p* = 0.010) on the involved are greater than the uninvolved limbs in group A, and ST (*p* = 0.010) has more muscle activation on the involved side than on the uninvolved side in group C (Figure 3).

### Gait Measurement Results

**FIGURE 4:**
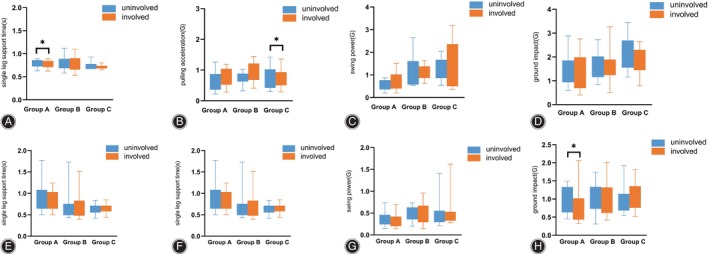
Single leg support time (A), pulling acceleration (B), swing power (C), and ground impact (D) for each group during going up the stairs. Single leg support time (E), pulling acceleration (F), swing power (G), and ground impact (H) for each group during going down the stairs.

For the gait parameters, we observed a difference in the comparison of single leg support time for the involved side than on the uninvolved side in group A (0.82 [0.72, 1.36] vs. 0.83 [0.71, 1.18], *p* = 0.009) and in the comparison of leg pulling strength in group C (1.03 [0.68, 1.21] vs. 0.75 (0.42, 1.02), *p* = 0.012) when going up the stairs. When going down the stairs, the ground impact on the uninvolved side of group A was greater than the ground impact on the involved side (0.90 [0.63, 1.33] vs. 0.71(0.43, 1.02), *p* = 0.035) (Figure 4A–H).

**FIGURE 5:**
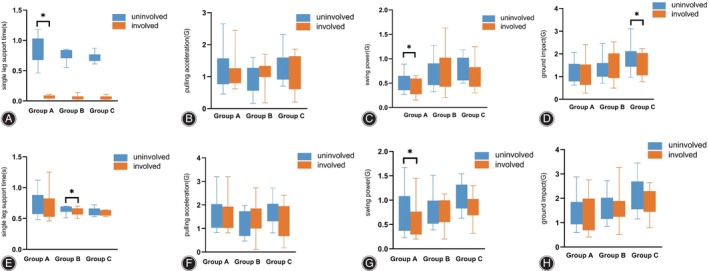
Single leg support time (A), pulling acceleration (B), swing power (C), and ground impact (D) for each group during normal speed walking. Single leg support time (E), pulling acceleration (F), swing power (G), and ground impact (H) for each group during fast walking.

When walking at normal speed, the single leg support time is greater on the uninvolved side than on the involved side in group A (0.78 [0.68, 1.03] vs. 0.72 [0.66, 1.00], *p* = 0.035). The swing power in group A [0.51 (0.36, 0.65) vs. 0.37 (0.27,0.59), *p* = 0.038] and the ground impact in group C [1.83 (1.43,2.12) vs. 1.39 (1.06, 2.04), *p* = 0.019] was greater on the uninvolved side than on the involved side. During fast walking, the swing power in group A (0.73 [0.37, 1.09] vs. 0.59 [0.30, 0.77], *p* = 0.044) and single leg support time in group B (0.66 [0.61, 0.70] vs. 0.61 [0.57, 0.67], *p* = 0.025) were greater on the uninvolved side than on the involved side (Figure 5A–H).

## Discussion

We found that for daily activities such as walking at normal speed, the quadriceps and hamstring muscles did not reach balance on the involved and uninvolved sides in the early stages (0–3 months) after reconstruction but did reach balance on both sides of the hamstrings at 4–12 months. At the same time, the quadriceps still had not fully recovered, while for movements that are more demanding on the knee joint, such as fast walking and going up and down the stairs, an imbalance was found for the hamstrings at 0–3 months and on the quadriceps at 4–12 months after reconstruction. However, for all these exercises, the balance of the RF on the involved and uninvolved sides was not achieved even 12 months after surgery. These findings have important implications for guidance at different stages of rehabilitation training. We also found that in the comparison between SLR (30°) and SLR (60°), SLR (30°) can activate more muscles; thus, SLR (30°) is a better training method than SLR (60°).

Biomechanical asymmetries in the gait process are very common and persistent after ACLR.^17^ The combined analysis of sEMG and gait is important, and gait reflects motor function and facilitates the assessment of the patient's prognosis.^18^ The analysis of sEMG includes time domain analysis and frequency domain analysis. Frequency domain analysis includes average power frequency and median frequency, which are used to reflect the local muscle fatigue level, while time domain analysis mainly includes integral EMG value, average EMG value, and RMS value, which mainly reflect the mobilization or recruitment level of local muscle motor units. Among them, the RMS value is often used to evaluate the activation level of muscles. Therefore, we chose RMS and normalized it for analysis.

### The Importance of Postoperative Rehabilitation

Postoperative rehabilitation is extremely important; good rehabilitation training is required to enable patients to quickly return to sports. However, the patterns of muscle atrophy after ACLR remain unclear, and an optimal rehabilitation training protocol has not yet been determined. Hiemstra *et al*.^19^ suggested that deconditioning, incomplete rehabilitation, and crossed‐nerve inhibition following ACLR can all lead to the bilateral lower extremity strength imbalance and that the presence of eccentricity over centripetal strength deficit in the uninvolved limb can lead to strength imbalance and may increase the probability of ACL rupture of the uninvolved side. Therefore, the goal of rehabilitation after ACLR is to restore the strength of the reconstructed ACL limb to be similar to that of the uninvolved limb. Hiemstra stated that assessing and restoring strength and power balance in the knee is an important objective component of ACLR and subsequent rehabilitation. Following ACLR, a period of training discontinuation may induce muscle atrophy. The disruption of proprioceptive information from the ACL can result in altered afferent inputs. Persistent knee pain and effusion may also lead to persistent injurious input. This altered afferent input may result in altered motor output, which can lead to specific force changes that can alter the strength balance of the knee in areas of strength deficit and joint strength balance. ACLR alters the neuromuscular balance of the knee joints. Correcting the imbalance in both lower extremities will not only improve functional outcomes but also prevent further injury.

### The Importance of Individualized Rehabilitation

With all of this in mind, an objective assessment of each individual patient's function in sports and its use as a basis to develop a rehabilitation program will be of great importance in the recovery of the patient's motor ability. In this field, numerous studies have confirmed that optimal gait stability can be achieved by balancing both lower limbs. Therefore, we believe that after reaching the quadriceps strength threshold, restoring strength alone may not improve gait asymmetry, and the effect of balance between the legs is more important. The quadriceps and hamstrings exert antagonistic forces on the tibia^20^; therefore, weakness of the extensor and flexor muscles of the affected limb is greater than that of the healthy limb after ACLR, and weakness in these muscles can affect knee stability, making it imperative to improve rehabilitation strategies to better target this outcome. Thomas *et al*.^21^ stated that lower extremity muscle weakness may affect dynamic lower extremity control and that identifying which muscles are weak is necessary so that rehabilitation strategies can precisely target the affected muscles. In some patients with increased muscle function but no recovery of motor function, it is necessary to confirm and quantify the presence of lower‐extremity muscle weakness after ACLR. Therefore, an objective assessment of the patient's function in sports and its use as a basis to develop a rehabilitation program will be of great importance in optimizing the recovery of the patient's motor ability.

### The Importance of the Characteristics of Muscle Activation and Gait Features at Different Times after ACLR

Our study found that the recovery in the flexor knee group was superior to that in the extensor knee group and that the rectus femoris did not completely recover for more than a year. White *et al*.^22^ previously found impaired quadriceps strength, abnormal movement patterns, and lower‐than‐normal knee function in patients following surgery. Buckthorpe *et al*.^23^ found that a considerable deficit in hamstring function was commonly found after ACLR in the early postoperative period, which is consistent with our findings. Inadequate knee flexor strength after ACLR leads to an increased risk of knee osteoarthritis, changes in gait and quality of motion, and an increased risk of reinjury after returning to sports. The loss of knee flexor strength is usually less than that of knee extensor strength. In a prior study, Sherman *et al*.^8^ found that individuals with ACLR exhibited higher hamstring sEMG amplitudes during gait and stair walking than healthy subjects and that weak individuals showed higher percent activation in tasks compared to MVIC relative to relative activation. Impairment of hamstring strength is prevalent in individuals after ACLR. This is similar to our results; however, our trial further determined the characteristics of muscle atrophy at different time points after ACLR.

### The Importance of Developing an Objective Quantitative Rehabilitation Program

Although many researchers have demonstrated the importance of muscle training in rehabilitation, an optimal training method has not yet been clearly defined. Erickson *et al*.^24^ found that blood flow restriction training may help improve persistent quadriceps strength loss associated with ACLR. Hasegawa *et al*.^25^, similarly concluded that electrical muscle stimulation implemented during the early rehabilitation phase was effective at maintaining and increasing muscle thickness and strength in the affected limbs. Jakobsen *et al*.^26^ found that because the early stages of knee injury rehabilitation focus primarily on mobility rather than high‐intensity strength training, elastic resistance exercises are recommended during this phase. Training the quadriceps disproportionately to the hamstrings may impair hamstring activation and decrease joint stability. Therefore, postoperative rehabilitation should also focus on the hamstrings to balance quadriceps activation in terms of muscle activation. The study of Khaiyat *et al*.^27^ showed that the anterior lunge and deep squat are the best exercises for the quadriceps, while the gluteus bridge and anterior lunge are the best exercises for the hamstrings. Ogrodzka *et al*.^28^ showed that neuromuscular electrical stimulation restores and improves quadriceps function and that this therapy helps activate the muscle. Meanwhile, White *et al*.^22^ found that neuromuscular training involving unstable perturbation of the involved and uninvolved lower extremities was a more effective means of enhancing function and reducing the risk of a second ACL injury after ACLR than strength training. Our study found that adopting an SLR of 30° was better for training than adopting an SLR of 60°.

## Limitation and Strengths

In this study, three different groups of subjects were used, and we did not follow up the patients long enough. We hope to improve this shortcoming in future studies by following up the patients for a long period of time, and provide more evidence to validate the results. The strengths of this study are as follows: (i) It confirms that sEMG and gait can be used to analyze the lower limb motor function of ACLR patients at different stages and to assess the patient's motor ability. (ii) The development of rehabilitation programs based on sEMG and gait is more conducive to the rehabilitation of patients.

## Conclusion

Imbalances in muscle function in both lower limbs have different manifestations over time. This study found that different muscles need to be focused on at different stages of the postoperative period. In the early stage of rehabilitation, the focus needs to be on the BF and ST; in the middle stage, it should be on the quadriceps, and in the late stage, it is the RF that needs to be focused on. sEMG and gait analysis can guide the development of a rehabilitation program, and the establishment of different individualized rehabilitation programs may enhance the effectiveness of training.

## Conflict of Interest Statement

The authors declare that they have no conflict of interest.

## Ethics Statement

This study was approved by the local ethics committee (Protocol 2022‐G29‐01). Written informed consent was obtained from all participants before the testing was performed.

## Author Contributions

Processing of the experiment, data analysis, and drafting the manuscript: Xipeng Wu. Revision of the manuscript: Hao Zhang and Hongxing Cui. Completion of the experiment and the statistical analysis: Wenbin Pei. Contribution to the process of the experiment: Yixuan Zhao. Assistance in the process of the experiment: Shanshan Wang. Design of the study and performance of the statistical analysis: Zhijie Cao. Conception of the study, designing and coordination, and helping to draft the manuscript: Wei Li. All authors have read and approved the final version of the manuscript and agree with the authors’ presentation order.

## Data Availability

All data generated or analyzed during this study are included in this published article.
